# ACNPD: The Database for Elucidating the Relationships Between Natural Products, Compounds, Molecular Mechanisms, and Cancer Types

**DOI:** 10.3389/fphar.2021.746067

**Published:** 2021-08-23

**Authors:** Xiaojie Tan, Jiahui Fu, Zhaoxin Yuan, Lingjuan Zhu, Leilei Fu

**Affiliations:** ^1^ School of Life Science and Engineering, Southwest Jiaotong University, Chengdu, China; ^2^ Key Laboratory of Structure-Based Drug Design and Discovery of Ministry of Education, School of Traditional Chinese Materia Medica, Shenyang Pharmaceutical University, Shenyang, China; ^3^ MOE Key Laboratory of Protein Sciences, Beijing Advanced Innovation Center for Structural Biology, Beijing Frontier Research Center for Biological Structure, Department of Basic Medical Sciences, School of Medicine, Tsinghua University, Beijing, China

**Keywords:** ACNPD, natural products, pharmacological mechanism, database, cancer

## Abstract

**Objectives:** Cancer is well-known as a collection of diseases of uncontrolled proliferation of cells caused by mutated genes which are generated by external or internal factors. As the mechanisms of cancer have been constantly revealed, including cell cycle, proliferation, apoptosis and so on, a series of new emerging anti-cancer drugs acting on each stage have also been developed. It is worth noting that natural products are one of the important sources for the development of anti-cancer drugs. To the best of our knowledge, there is not any database summarizing the relationships between natural products, compounds, molecular mechanisms, and cancer types.

**Materials and methods:** Based upon published literatures and other sources, we have constructed an anti-cancer natural product database (ACNPD) (http://www.acnpd-fu.com/). The database currently contains 521 compounds, which specifically refer to natural compounds derived from traditional Chinese medicine plants (derivatives are not considered herein). And, it includes 1,593 molecular mechanisms/signaling pathways, covering 10 common cancer types, such as breast cancer, lung cancer and cervical cancer.

**Results:** Integrating existing data sources, we have obtained a large amount of information on natural anti-cancer products, including herbal sources, regulatory targets and signaling pathways. ACNPD is a valuable online resource that illustrates the complex pharmacological relationship between natural products and human cancers.

**Conclusion:** In summary, ACNPD is crucial for better understanding of the relationships between traditional Chinese medicine (TCM) and cancer, which is not only conducive to expand the influence of TCM, but help to find more new anti-cancer drugs in the future.

## Introduction

Cancer, with the continuously increasing morbidity and mortality, is the second most deadly disease in the world, which seriously threaten people’s life and health. (Fidler et al., 2017). At present, continuous proliferation, invasion and metastasis, angiogenesis and apoptosis resistance have been founded to be closely related to the occurrence and development of cancer (Hisano and Hla, 2019; Wimmer et al., 2020). For example, tumor metastasis is a common process in the development of malignant tumors, in which cancer cells undergo the invasion–metastasis cascade, including five main steps: leaving the primary lesion, passing through blood vessels, spreading through blood circulation and lymphatic circulation, or directly spreading through body cavity, continuing to proliferate and grow in other parts, and eventually forming colonization at a remote site of the body (Mittal, 2018). In this process, occurred autophagy and apoptosis inhibition may equip cancer cells with enhanced ability to divide and proliferate (Dower et al., 2018). In addition, abnormal metabolic and immune escape, as well as changes in the tumor microenvironment, are also considered to be crucial hallmarks of cancer (McGranahan et al., 2017; Counihan et al., 2018; Hinshaw and Shevde, 2019). Currently, many kinds of clinical treatments, including surgery, chemotherapy, radiotherapy, immunotherapy and gene therapy have been applied to treat cancer. (Fesnak et al., 2016; Chen and Mellman, 2017; Chen et al., 2019). However, the limited application scope and severe side effects restrain of above methods make the use of anticancer drugs crucial in treating cancer.

Notably, natural products (referring to natural compounds derived from Traditional Chinese Medicine (TCM) plants and derivatives are not considered herein) constitute an important source of anti-cancer drugs, and about 50% of the anti-cancer drugs currently in use are directly or indirectly derived from natural products, including flavonoids, alkaloids and terpenoids and others (Mondal et al., 2019; Deng et al., 2020; Kopustinskiene et al., 2020). Over the past two decades, a large number of natural anti-cancer products have been extracted, isolated and purified from Chinese herbal medicines. For example, paclitaxel, extracted from the bark of *Taxus chinensis*, is recognized as one of the most potent and broad-spectrum anti-cancer drugs especially for advanced, metastatic ovarian cancer, breast cancer, lung cancer (Abu Samaan et al., 2019; de Goeje et al., 2019; Zhu and Chen, 2019; Lau et al., 2020). Recently, accumulating evidences have shown that compounds derived from natural products could regulate various targets and signaling pathways and displayed great therapeutic potential in cancer. Curcumin, a polyphenolic compound mainly extracted from *Curcuma longa, Curcuma zedoaria and Acorus calamus* L., has been shown to induce autophagy through 5'AMP-activated protein kinase (AMPK) activation, resulting in the degradation of Akt, thereby inhibiting cell proliferation and migration of human breast cancer MDA-MB-231 cells (Guan et al., 2016). Additionally, curcumin induces apoptosis and autophagy in human non-small cell lung cancer A549 cells by inhibiting the phosphatidylinositol 3 kinase (PI3K)/Akt/mammalian target of rapamycin (mTOR) pathway (Liu et al., 2018). Berberine, an isoquinoline alkaloid, induces autophagic cell death in cancer cells by increasing the level of Glucose regulated protein 78 (GRP78) and enhancing its ability to bind to vacuolar protein sorting 34(VPS34) (La et al., 2017). Berberine also inhibits the epithelial mesenchymal transformation of mouse melanoma B16 cells through PI3K/Akt pathway (Kou et al., 2016). Moreover, ursolic acid, isolated from Spreng (bearberry), *Rhododendron hymenanthes* Makino, *Eriobotrya japonica*, etc., plays an anti-cancer role in human ovarian cancer cells through apoptosis induction, cell cycle arrest and down-regulation of PI3K/AKT pathway, which is mediated by reactive oxygen species (Ros) and matrix metalloprotein (MMP). Interestingly, monomer compounds isolated from natural products can also be used as lead compounds for the synthesis of anticancer drugs. Through structural modification, they eventually play an important role in the treatment of many malignant tumors such as breast cancer, cervical cancer and leukemia, and greatly promote the development of new candidate drugs. (Rodrigues et al., 2016; Spradlin et al., 2019; Wang et al., 2019; Patel et al., 2020).

Currently, a few traditional Chinese medicine web servers are available online to facilitate resource search, such as and Traditional Chinese Medicine database (TCM-database) and Traditional Chinese Medicine Systems Pharmacology database and Analysis Platform (TCMSP). However, few databases of natural anti-cancer products have been constructed, and the existing databases only constitute the framework of active ingredient-targets-disease, while no databases holds a systematic summary of the specific mechanism of natural products against cancer. (Zhang et al., 2019). Thus, we have constructed an online database, ACNPD, focusing on the information of compounds (referring to natural compounds derived from Traditional Chinese Medicine (TCM) plants) and their pharmacological mechanisms. ACNPD is expected to help deepen the understanding of the intricate molecular mechanism of natural products in treating cancer, with a view to providing new clues for the development of anticancer drugs.

## Materials and Methods

### Data Collection and Integration

ACNPD is a collection of anti-cancer compounds in traditional Chinese herbal medicine, which can be divided into chemical and pharmacological parts, especially focusing on the anti-cancer molecular mechanism of natural products. Markedly, considering the variety and complexity of natural products, the pharmacological mechanisms of the compounds in the database are supported not only by systematic *in vivo* and *in vitro* studies, such as targets, signaling pathways, validation of animal models, etc., essential *in vitro* cytotoxicity tests are also included. These information and data come from the related network database and the text mining of the published articles. The names of natural products, IUPAC names, SMILES and CAS numbers were obtained primarily by PubChem (https://pubchem.ncbi.nlm.nih.gov/) and supplemented and verified by ChemicalBook (https://www.chemicalbook.com/) and Reaxys (http://new.reaxys.com). The TCMSP database (http://tcmspw.com/tcmsp.php), the Chemical composition database of natural products in China (pharmdata.ncmi.cn/cnpc/) are used to search and collect herbal sources of natural anticancer products. We retrieved and sorted the pharmacological information of the compounds from a series of databases such as PubMed, SciFinder and Web of Science, and finally integrated them into six categories: inhibition of proliferation, promotion of apoptosis, autophagy, necrosis, inhibition of invasion and molecular mechanism. In brief, ACNPD is a comprehensive, high-quality, freely available database of natural products against cancer (Table 1).

**TABLE 1:** Data source.

Data field	Data source	Amount of data
Natural compounds	ChemicalBook, PubMed, PubChem, SciFinder and Reaxys	521
Mechanisms	PubMed, SciFinder and Web of Science	1,593
Diseases	PubMed, SciFinder, TCGA, Text-mining and Web of Science	10

### Web Server Generation

ACNPD builds Apache network server (version 2.4, The Apache Software Foundation, Wakefield, MA, United States) based on Linux operating system, PHP (Versions 7.1, Zend Technologies, Cupertino, CA, United States) scripting language for MySQL (version 5.1.48-log, Oracle Corporation, Redwood Shores, CA, United States). As the background system of ACNPD database, the system or software used is not only free and open source, but also has good stability and high security. The database interface is built on Web technology, Including CSS (Cascading Style Sheets), HTML (Hypertext Markup Language) and JavaScript (Oracle Corporation, Redwood Shores, CA, United States). Adopt Web server technology to develop and serve Web applications. Currently, the ACNPD database supports retrieval by all major Web browsers.

### Web Server Construction and Structure

The integrated anti-cancer natural product data is composed of two main EXCEL tables, which are basic chemical information and pharmacological mechanism, respectively. Name, IUPAC Name, SMILES, CAS number, and Herb source of each compound are included in chemical information. The IUPAC Name is the Name of the International Union of Pure and Applied Chemistry, standardizing the names of all compounds in order to convert them into structural formulas corresponding to natural products. Importantly, information about pharmacological mechanism is the focus of our work, which is classified into six categories, including inhibition of proliferation, induction of apoptosis, autophagy, necrosis, inhibition of invasion. The above data and information are obtained from the network database and text retrieval, with guaranteed accuracy. Of note, the two datasheets can be interchanged by hyperlinking to meet different retrieval needs of users.

## Results

### The Extracted Information of ACNPD

**FIGURE 1:**
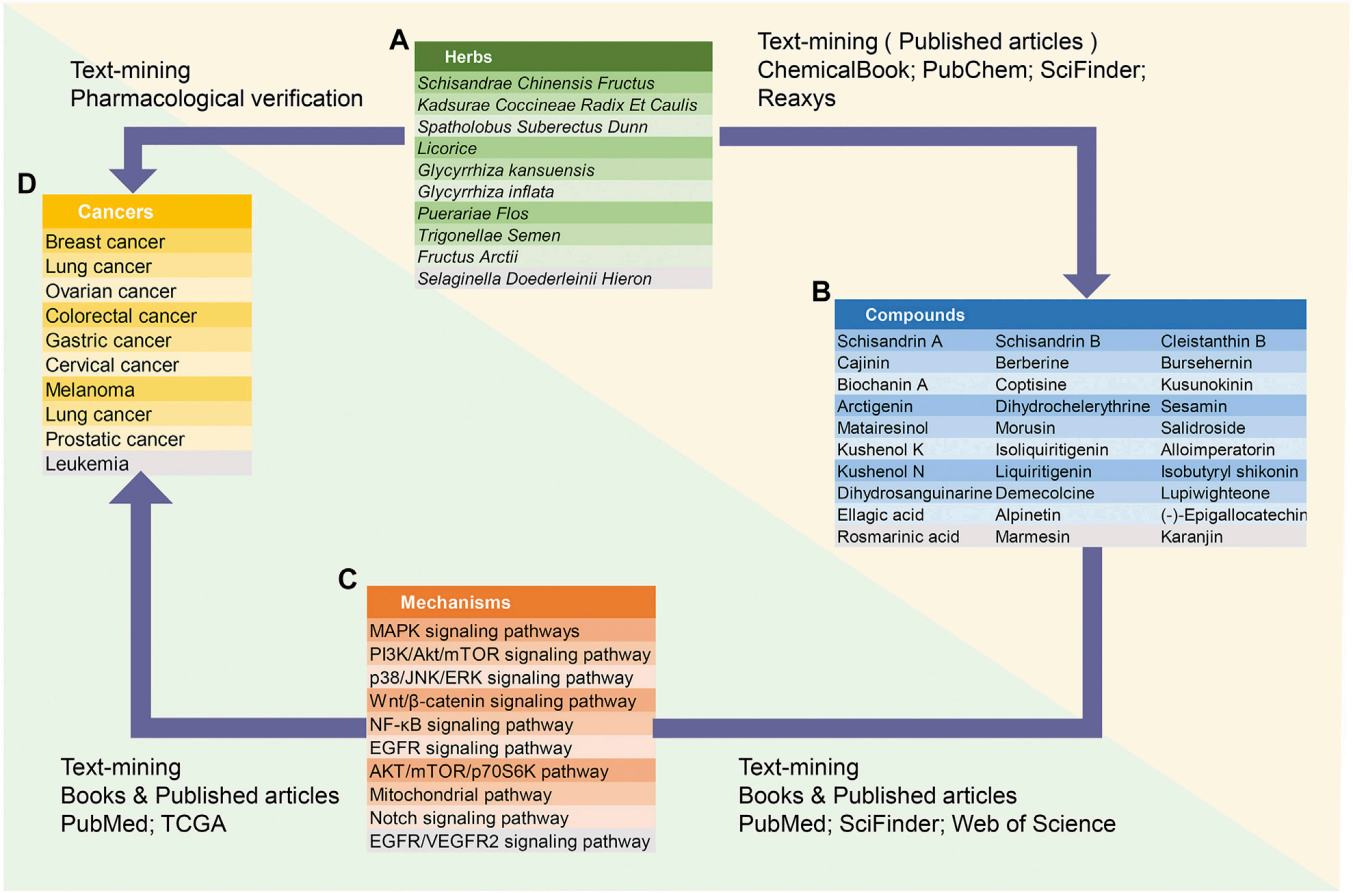
Database structure. ACNPD is composed of compounds, traditional Chinese medicine sources, cancer types, and regulatory mechanisms. The relationship between the four key modules in ACNPD are described. Notably, the yellow triangle area represents the basic chemical information and the green triangle area represents the pharmacological mechanism contents.

**FIGURE 2:**
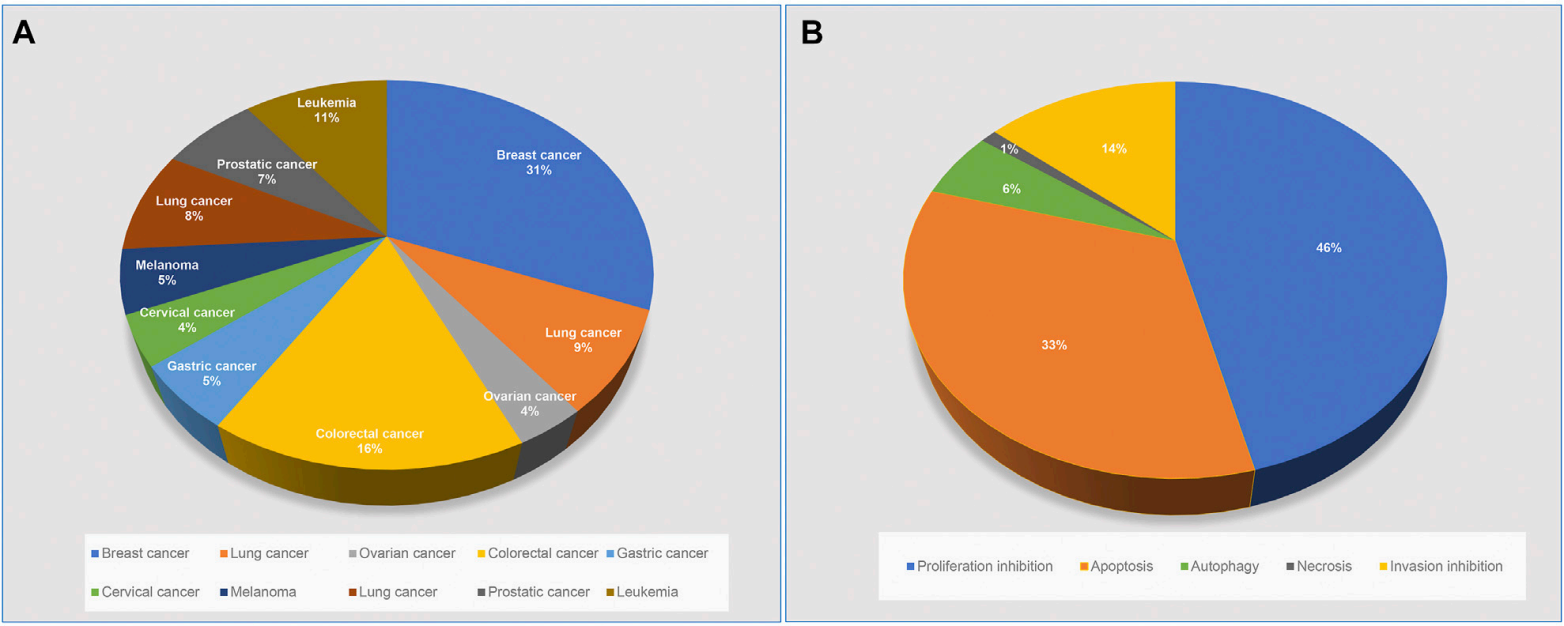
Classification of compounds in the ACNPD. **(A)** Classified by cancer types: 521 compounds derived from natural products were involved in 10 cancer types and percentages, including: Breast, cervical, ovarian, lung, gastric, liver, colorectal, prostate, leukemia and melanoma. **(B)** Classified by pharmacological mechanisms: all compounds are classified into five groups according to text retrieval: proliferation inhibition, apoptosis, autophagy, necrosis, invasion inhibition. Note: Groups are marked with different colors. The percentage of compounds contained in each group is shown in the pie chart.

ACNPD brings together the chemical information and pharmacological mechanism of anti-cancer natural products to elucidate the intricate relationship between anticancer active compounds in herbs and cancers (Figure 1). Interestingly, we searched related literatures in PubMed, SciFinder and Web of science by text-mining, and classified the pharmacological contents in detail, focusing on the related target proteins and pathways. By contrast, given the large number of existing databases of compounds, such as PubChem, ChemicalBook and others, the chemical information of natural products in ACNPD provides only basic information, including the IUPAC Name, SMILES, CAS number, and herbal source. Hitherto, a wide variety of cancers have been uncovered. Focusing on the statistical data published by the World Health Organization's International Agency for Research on Cancer (IARC), considering morbidity and mortality, the final 10 cancer types were determined to be included in ACNPD, such as breast, cervical, ovarian, lung, gastric, liver, colorectal, prostate, leukemia, and melanoma. In addition, the 13 common cancers provided by The Cancer Genome Atlas (TCGA) database and the literature search results were also included as a reference condition (Supplementary Table S1) (Figure 2). Currently, the database contains 521 compounds and most of them show anti-cancer activity against a variety of cancers. For example, berberine, an alkaloid that isolated from herbs such as *Achyranthis Bidentatae radix*; *chelidonii herba*; *Coptidis Rhizoma*, etc., displays activity against 10 cancer cells mentioned above (Zhao et al., 2017; Mohammadlou et al., 2021). Studies have shown that berberine inhibits proliferation of colon cancer cells by inhibiting the Wnt/β-catenin signaling pathway (Wu et al., 2012). In another study, Berberine was found to inhibit TNF-α-induced matrix metalloprotein 9 (MMP-9) and cell invasion through inhibiting activating protein-1 (AP-1) in MDA-MB-231 human breast cancer cells (Kim et al., 2008). Additionally, 153 compounds such as Licoricidin, Limonin and Wogonin have been found to have anti-cancer effects by affecting the corresponding signaling pathways, mainly involving the Signal Transducer and Activator of Transcription 3 (STAT3) signaling pathway, PI3K/Akt/mTOR signaling, Wnt/β-catenin signaling pathway (Rasul et al., 2012; Ji et al., 2017; Chen et al., 2018). Compounds in ACNPD were subdivided into five categories according to different pharmacological mechanisms, among which 262 compound induced programmed cell death and 109 compound inhibited cell invasion.

### Database Query

**FIGURE 3:**
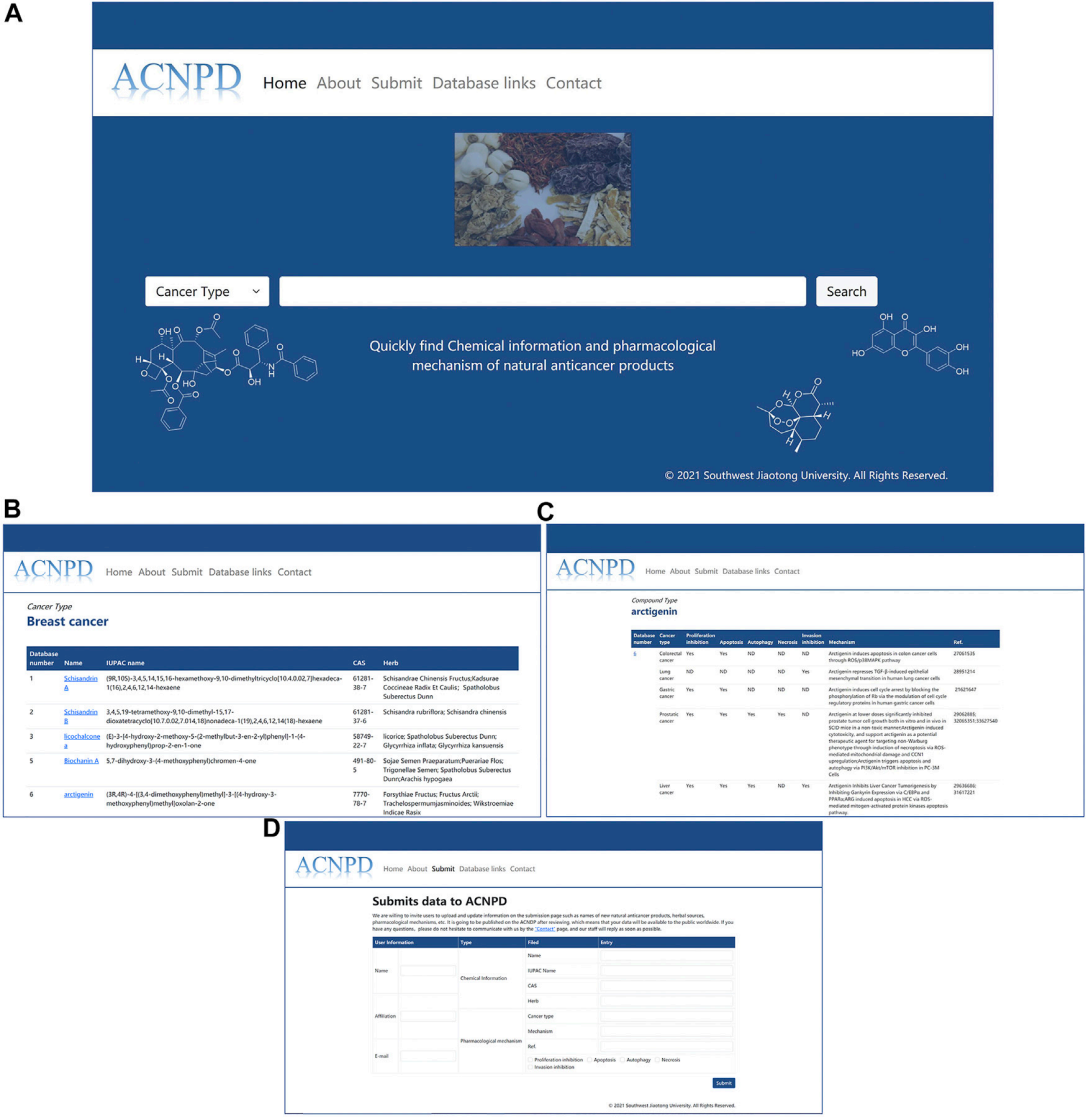
Interface of ACNPD website. Users can search the database in two ways and query the detailed information through a link. For example, users can query not only by entering a cancer type name (such as Breast cancer) as a keyword, but also by entering the name of a compound from a natural product for field retrieval (e.g., arctigenin). **(A)** Query Home Page; **(B)** Breast cancer Search Result; **(C)** Search Result of Compound Arctigenin; **(D)** Submit Interface.

We built a friendly web interface that allows users to easily search across multiple browsers. Users can obtain the desired results through fuzzy search as follows: 1. Enter the type of cancer (currently limited to the 10 mentioned above), and the results page displays the relevant natural product items with the links to access the basic chemical information and pharmacological mechanism. For example, users can query 164 compounds with anti-breast cancer activity by entering breast cancer. Click the desired compound arbitrarily, and the corresponding database number, Herb source, pharmacological activity and other information will be displayed. 2. Users can use ACNPD to query the mechanism information of natural products. For example, by entering the name of a compound or CAS number, users can obtain the corresponding anticancer mechanism of this compound and intuitively know whether the natural product can inhibit proliferation, inhibit invasion, and whether it is related to autophagy. The relevant content can be verified by the references we provide for more detailed information. (Figure 3).

## Discussion

Cancer, also known as malignant tumor, is characterized by cells with abnormal proliferation, which is caused by mutated genes generated by external and internal factors. Hitherto, malignant tumors have been threatening human health in modern society. Recently, with the continuous upgrading of tumor biology and the emergence of drug problems such as anti-cancer drug resistance in clinical use, it is urgent to develop novel anti-cancer drugs. Notably, natural products, with their potent anti-cancer effectiveness and abundant resources, have become the top priority of anti-cancer drug research and development.

Accumulating evidence has indicated that small-molecule compounds derived from natural plant products inhibit cell proliferation, metastasis, and invasion, regulate cell cycle, and induce cell apoptosis in various types of cancer through different targets and signaling pathways. For example, a large group of natural products, flavonoids, display pro-apoptosis effect, with Genistein, which acts on the p53 non-apoptotic pathway, and quercetin, which acts on the cytochrome C pathway. (Safarzadeh et al., 2014; George et al., 2018). Resveratrol inhibits the metastasis of cancer cells by down-regulating the expression of MMP-2, 9 by inhibiting the c-Jun N-terminal kinase (JNK) pathway (Bae et al., 2017). Quercetin, shikonin, and nobiletin are also demonstrated to inhibit cancer metastasis through suppressing MMP. (Jang et al., 2014). In addition, many compounds, including evodiamine, oridonin and jaceosidin, act on the PI3K/Akt pathway, which is a common inflammatory and cancer transformation pathway. Phosphorylation of its substrate GSK-3β can inhibit the translocation of Bax and the degradation of Bcl-2, leading to apoptosis inhibition of cancer cells (Mohan et al., 2016; Woo and Kwon, 2016). Arctigenin, a lignanolids isolated from *Arctium lappa* L. inhibits the angiogenesis through downregulating MMP-2, MMP-9 and heparinase (Lou et al., 2017). In recent years, emerging research suggested that the number and diversity of therapeutic targets in cancer far exceeds the oncogenes identified so far, expanding the understanding of cancer biology and corresponding therapeutic strategies (Hahn et al., 2021). In addition to traditional anti-cancer targets, natural products have also been found to act on several emerging targets, including targeted inhibition of histone deacetylase (HDAC), targeted inhibition of heat shock protein HSP90, targeted glycolysis, and others (Palermo et al., 2005; Yoon and Eom, 2016). Although natural products have unique anti-cancer properties, there is a lag in the construction of relevant mechanism databases, thus, the development of ACNPD has broad application prospects and far-reaching practical significance.

Importantly, ACNPD is an online webserver that provides users with the pharmacological mechanisms of natural anti-cancer products. The database contains 10 common cancer types, 521 compounds, 131 pharmacological pathways, including PI3K/Akt, STAT3, PI3K/Akt/mTOR, Wnt/β-catenin and other signaling pathways. The web interface allows users to query related information in a variety of ways, providing basic chemical information, such as IUPAC Name and CAS number, and detailed pharmacological mechanisms of natural compounds. Importantly, we divided the pharmacological effects in a more detailed way and provided the signaling pathways involved, which is a supplement to the previous database and helps users to obtain information quickly and intuitively. At present, ACNPD only contains 10 common cancer types with high incidence, and the number of compounds remains to be expanded. With the extraction and separation of more natural products and the in-depth study of their pharmacological mechanism, we will continue to expand the scope of cancer and add new natural compounds to update ACNPD.

In summary, we believe that ACNPD is a valuable database to fill the gaps between the therapeutic mechanism and natural anti-cancer products. The database is expected to help study the biological function and pharmacological activity of natural products in cancer, break the development bottleneck between natural anticancer compounds and modern anticancer drugs, and usher in a promising prospect for the innovative anticancer drugs.

## Data Availability

The original contributions presented in the study are included in the article/Supplementary Material, further inquiries can be directed to the corresponding authors.

## Abbreviations

ACNPD, Anti-cancer natural product database; AP-1, activating protein-1; GRP78, Glucose regulated protein 78; HDAC, Histone deacetylase; IUPAC, International Union of Pure and Applied Chemistry chemical nomenclature; JNK, c-Jun N-terminal kinase; mTOR, mammalian target of rapamycin; MMP, matrix metalloprotein; MMP-9, Matrix metalloprotein 9; PI3K, phosphatidylinositol 3 kinase; ROS, Reactive oxygen species; STAT3, Signal Transducer and Activator of Transcription 3; TCGA, The Cancer Genome Atlas; TCM database, Traditional Chinese Medicine database; TCM, Traditional Chinese medicine; TCMSP, Traditional Chinese Medicine Systems Pharmacology database and Analysis Platform; TNF-α, Tumor necrosis factor-α; VPS34, vacuolar protein sorting 34.
