# Supplementary material for: ACNPD: The Database for Elucidating the Relationships Between Natural Products, Compounds, Molecular Mechanisms, and Cancer Types
Source: Front Pharmacol. 2021 Aug 23;12:746067. doi: 10.3389/fphar.2021.746067 (PMC8419280; doi:10.3389/fphar.2021.746067)
Supplement: Supplementary file 1 [file Table1.docx]

**Table S1 The Global Cancer Statistics in 2020**

| **Cancer type** | [**Incidence**](javascript:;) | [**Mortality**](javascript:;) |
| --- | --- | --- |
| Breast cancer | 11.7% | 6.9% |
| Cervical cancer | 3.0% | 3.4% |
| Ovarian cancer | 1.6% | 2.1% |
| Lung cancer | 11.4% | 18.0% |
| Gastric cancer | 5.6% | 7.7% |
| Liver cancer | 4.7% | 8.3% |
| Colorectal cancer | 9.7% | 9.4% |
| Prostate cancer | 7.3% | 3.8% |
| Leukemia | 2.5% | 3.1% |
| Melanoma | 1.7% | 0.6% |

*All data are from the World Health Organization's International Agency for Research on Cancer (IARC) Global Cancer Statistics 2020.
